# Caring for quality of care: symbolic violence and the bureaucracies of audit

**DOI:** 10.1186/s12910-015-0006-z

**Published:** 2015-05-08

**Authors:** Nathan Emmerich, Deborah Swinglehurst, Jo Maybin, Sophie Park, Sally Quilligan

**Affiliations:** School of Politics, International Studies and Philosophy, Queen’s University Belfast, Belfast, UK; Barts and The London School of Medicine and Dentistry, Queen Mary, University of London, London, UK; The King’s Fund, London, UK; Research Department of Primary Care and Population Health, UCL Medical School, London, UK; University of Cambridge, School of Clinical Medicine, Cambridge, UK

**Keywords:** Care, Quality of care, Ethics of care, Bourdieu, Symbolic violence, Audit

## Abstract

**Background:**

This article considers the moral notion of care in the context of Quality of Care discourses. Whilst care has clear normative implications for the delivery of health care it is less clear how Quality of Care, something that is centrally involved in the governance of UK health care, relates to practice.

**Discussion:**

This paper presents a social and ethical analysis of Quality of Care in the light of the moral notion of care and Bourdieu’s conception of symbolic violence. We argue that Quality of Care bureaucracies show significant potential for symbolic violence or the domination of practice and health care professionals. This generates problematic, and unintended, consequences that can displace the goals of practice.

**Summary:**

Quality of Care bureaucracies may have unintended consequences for the practice of health care. Consistent with feminist conceptions of care, Quality of Care ‘audits’ should be reconfigured so as to offer a more nuanced and responsive form of evaluation.

## Background

The delivery of ‘care’ is at the core of any system of health care, something that we understand to be constituted by a complex network of interdependent practices that can be seen as being ‘ordered’ within the micro, meso, and macro social contexts of care: delivery, management and political oversight^b^. The idea(l) of ‘care’ is an essentially normative or moral concept; arguably to care badly is not to care at all. There is a practical and moral obligation on health care organizations to manage the delivery of ‘care’ or, perhaps more importantly, to ensure services are provided *with care*. Health and social care services encompass activities as wide ranging as bathing or feeding a patient to the deployment of complex medical technologies and medicines^c^. Whilst these diverse activities contribute to constituting health care services, it is in the *manner* of their delivery and the particularities of their *provision* that the essence of ‘care’ is to be found. As Mol argues, the ideal of good care does not speak for itself as it is not constituted by the services themselves; rather, it is something that ought to be incorporated into such services [1]. Care can be thought of as residing in the subjective, and intersubjective, experiences of patients, clients and professionals. That is, care is not merely an attribute of a particular service but the way it is *provided* or *delivered*. Care involves an emotional stance and relational quality that can, but may not, accompany the activities constitutive of health care provision.

In this view, taking care of someone involves not only being careful in the sense of proceeding with due attention but involves *being* ‘care’-ful, in the sense of adopting or embodying a particular emotional posture or stance [2]. Given the dual nature of ‘care’ in health care – it is both service and stance - the meaning of the phrase ‘Quality of Care’^a^ is, in some sense, a second order idea which combines these two components. It is not usually about the services and the delivery of care *per se*, but about ways in which services are organized, delivered and *accounted for*^d^ in response to particular organizational structures or bureaucratic regimes. The scale we invoke by considering the notion and operationalization of Quality of Care is not the micro-level of practice or the interaction between professional and patient, (where care is actually experienced in all its intersubjective nuance) but the meso- and macro- level of social structure - the social organization of care and its institutionalized control. Quality of Care operates at the level of the clinic, the hospital, the NHS, the Care Quality Commission and broader socio-political arrangements. Furthermore, Quality of Care emerges from a particular orientation towards our system of health care, an orientation which collectivizes, generalizes and - to a large extent- decontextualizes care itself. Thus we suggest contemporary approaches to the care and the management of care reiterate the persistent tendency to eliminate the messy business of ‘care itself’ from the public sphere. The inherently (inter)subjective experiences of care and caring, the ‘lifeworld’ of individuals and small groups of individuals participating in the *actual* provision of care are relegated to the private realm whilst only objectified measures of its ‘quality’ are afforded ‘public’ - managerial or political - significance.

## Discussion: care and quality of care

It is clear that one cannot conceive of a system of health care that does not involve the delivery of care. The practical tasks such systems deliver are diverse and range from the mundane, day-to-day and routine to the unusual, ‘high tech’ and singular. Consider, for example, the following examples: getting people out of bed, washing them, providing food, ensuring medicines are taken at the correct time and dose, providing physiotherapy or rehabilitation services, monitoring life signs, conducting tests (urine, blood, radiographic etc.), providing vaccinations or intravenous infusions, undertaking internal examinations, conducting operations and transplanting organs. All can be considered examples of patient ‘care’ and we might, therefore, wonder how to define care such that it is applicable to this range of activities. We might think of ‘care’ as a secondary and normative quality of first order tasks or substantive practices. Whether done badly or well, getting someone up in the morning or providing a program of rehabilitation is to provide care. However when it is done well it is done *with* care. It is this sense of care that is of primary interest, and something that is present in Tronto’s suggestion that “[c]are is both a practice and a disposition” [3]. In her view we should only refer to care when both are present. Thus, the nurse who washes patients in an uncaring manner does not provide care in the fullest possible sense, even though such activities can be considered as care *simpliciter*.

Tronto and Fisher define care as:

[A] species of activity that includes everything that we do to maintain, continue, and repair our ‘world’ so that we can live in it as well as possible [3,4]^e^.

So defined, care is a disposition to ‘living well’ that can be expressed in almost all activities. As such care almost always occurs as part of other activities. Whilst some activities instantiate care alone as in, for example, a parent who comforts their child when they have grazed their knee or a spouse who reassures their partner before their first day in a new job. Even so, these caring acts can be considered as actions being done with care. This being the case there is clearly an ethical and normative dimension to care. We might connect this to the sociological fact that a profession instantiates an ethics or, better, an *ethic* [5,6]. A professional is someone who has made a moral commitment to their clients, someone who places the interests of their clients above their own. When considered alongside the concept of a professional ethics, Tronto’s notion of care makes explicit the double normativity of the caring professions. As a recent (2014) World Nursing Day slogan put it: “Nursing is my skill; Caring is my profession”. There is a normative dimension to the intersubjective practice of care-giving as well as in the more ‘skills focused’ formal ethics of the caring professions, and these dimensions may not be aligned in all cases. Health care professionals do not simply ‘profess’ their training or expertise; they profess to care. This should not be read in the sense that they *say* they care but in the sense that they pursue their profession as a means of caring. The slogan speaks to the moral motivation, both initial and ongoing, of those who join the caring professions. As Tronto puts it “caring is not so much about the activities of care, but about the emotional investment that has been made in order to care” [3]^f^.

Given such a perspective we might consider the question of how these ideals – those of individual professionals, of professions as social institutions, and of the philosophical characterization of care - connect with the realities of practice and the practical delivery of care. First, we might note that they often do not. Recent failures in NHS care been extensively documented but are not necessarily a new phenomenon [7]. In the 1960’s Menzies Lyth’s seminal article ‘Social systems as a defense against anxiety: An empirical study of the nursing service of a general hospital’ articulated the difficulties inherent in care as an everyday occupation, and the consequences for its social organization [8]^g^. In her work Lyth details some of the emotional challenges that arise within the practice of nursing – intimate contact with strangers, the uncertainty of recovery, the distress associated with chronic illness – and how a task-based, rather than patient centered division of labor provide for a degree of organizational defense or structural distance.

More recently Mol has analyzed what she calls the ‘logic of care’ [1]^h^. As an aspect of the logic of practice, care always co-exists with other - sometimes competing or incommensurable - logics [9]. Part of Mol’s purpose is to contrast the ‘logic of care’ with the ‘logic of choice’ and to examine their uneasy relationship [1]. However, she also suggests the logic of care co-exists with “neglect and errors”, things that are, unfortunately, ineliminable parts of everyday life [1]^i^. Mol’s analysis suggests that ‘care’ cannot be used to define what is ‘good’, ‘better’ or ‘worse’, at least, not outwith the logic of care, i.e. the actual practice(s) of health care [1]. ‘The good’ is defined within such practices and what care *is*, is the situated pursuit of ‘the good’ according to the contextually relevant logic(s). Care is part of the situated pursuit of the good, it is part of this species of activity. Attempts to define the good (or what constitutes care in a particular instance) cannot “precede practice, but form a part of it” [1]. Thus, “[t]he good is something to *do*, in practice, as care goes on” [10] and the practices of care “entail a specific *modality* of handling questions to do with the good” [10].

This view might be taken as being in conflict with the fact that Tronto does define care. However, her suggestion is that we consider care to be “a species of activity that includes everything that we do to maintain, continue, and repair our ‘world’ so that we can live in it as well as possible” [3]. In this view *good* care or, we might say, *quality* care is marked by “persistent tinkering in a world full of complex ambivalence and shifting tensions” [10]. Her definition of care suggests that what care *is* needs to be determined in practice and according to context. However, in recent times the notion of ‘quality’ and not simply ‘good’ care has become politically significant and embedded in the governance of modern health care. In contrast to the idea of an object’s ‘property’ (or, to use the philosophical term, its *predicates*) - but like care - the notion of an object’s *quality* is (like the notion of ‘care’) inherently normative. Contemporary use of the word ‘quality’ in the context of care assumes that we can sensibly talk about quality in the abstract. This is misguided. If a discussion of quality is to be useful it must refer to particular historically and socio-culturally situated practices. A definition of quality, independent of such context, is next to useless. Arguably, then, a concern for the ‘Quality of Care’ can be taken to be a concern for a level of excellence in health and social care. Concern for the standard(s) of practice has been translated into concern for not just the assessment, audit and evaluation of those standards, but a particular form or approach to ‘quality assurance’. In practice any attempt to conduct ‘quality assurance’ assessments will be embedded in specific managerial and bureaucratic processes^j^. Such processes are the primary mode through which health care is evaluated and governed, rendering them subject to public and *political* scrutiny. As a consequence these processes have reflexive implications for the social organization of practice, a point we discuss further below. They also require some kind of ‘operational’ definition: whilst the notion of *care* resists definition, Quality of Care cannot.

What we might take Quality of Care to mean is open to contestation and debate. It resists simple *a priori* definition and is always in need of contextual operationalization. Insofar as particular contexts are comparable, or have common (social or structural) features, such as those contexts within which various health care practices are to be found, Quality of Care has been afforded a certain degree of characterization. Goldenberg considers attempts to provide such characterization a ‘catalogue approach’, the main virtue of which is that it “helpfully provides at least some guidance for policy and allocation purposes” [11]. However, she argues that the various traits and characteristics that are catalogued for the purposes of defining Quality of Care are not accompanied by a detailed justification. Instead they are understood to be aligned with the prevailing “norms and values currently guiding (ideal) health care practice” [11].

Goldenberg’s broader argument is that this ‘catalogue’ approach means that Quality of Care is ‘persuasively’, and therefore *politically*, defined. Such definitions are predicated on the surrounding discourses through which they derive constitutive meaning, the discourses of public management, bureaucracy, and governance being particularly influential. In providing a ‘headline’ definition of Quality of Care these discourses must also provide for a level of flexibility in order that the existing variety of services can be evaluated. As such, any definition of Quality of Care is useful not because of the detail it provides but because that detail reflects the emotive, evaluative or rhetorical content of the term. If a definition is to be useful it will leave the rhetorical or *political* aspects of the term intact [11]. However, whilst any attempt to define ‘Quality of Care’ will contain ambiguous elements and whilst it will always remain possible to object to particular, ‘catalogued’, details, it is nevertheless the case that general claims about the importance of ‘quality care’ will remain difficult to resist. Consequently the management and governance of health care will continue to need and rely on ‘Quality of Care’ discourses. The *idea* of Quality of Care has a *political* function, one that can be used to engender the formation of social movement(s) and promote change. However the idea also aligns with the socio-political context of contemporary governance, the management of public services and with the specific ‘organizational format’ of its associated bureaucracy^k^.

In the context of health care, ideas about Quality of Care can be placed in the service of a variety of ends, not all of them compatible with each other. For example, we might think Quality of Care governance acts to improve care itself or acts as a device that mitigates political accountability by institutionalizing a bureaucratized form of managerial accountability. In so doing ministers are afforded a degree of protection since although they remain answerable they are no longer directly accountable [12]. If we think political accountability is important then, as Nelson remarks, “obscurities concerning the notion of ‘quality of care’ are not matters about which we can afford to be insouciant” [13]. We might then conclude that the obscurities we should be concerned with are not simply internal but also relate to the broader political role of the notion and its institutionalization. A definition offered by Goldenberg is useful precisely because it highlights the potential for internal and external conflicts, the variation in situated understanding, and the perspectival contestations inherent in the meaning of Quality of Care. She says that:

Quality of care refers to the attributes of a health care service that are taken by the relevant stakeholders to be important enough to be measured and promoted within an organization [11].

With deliberate irony, Goldenberg goes on to suggest that this is “the kind of definition that should not be entertained in the health quality assurance literature” because: it offers “no logical room for dissent”; it avoids “all potential points of disagreement by taking no position on whether the term refers to process or outcome, property or evaluation”; it does not imply any “potentially controversial position on the content or context of quality care”; and the vacuity of this “descriptive meaning [means that a wide variety of meanings or interpretations] can be carefully attached to the term by savvy policy makers” [11]. Whilst we agree with Goldenberg that the flexibility of this definition is deeply problematic in practical or political terms it is nevertheless particularly useful for the purposes of critical analysis of Quality of Care discourses and the practices that are meant to ensure it. The analytic utility of this definition lies in its potential to highlight the fact that the ability to what is important enough to be measured and promoted within particular contexts is *politically determined*; it is a function of power. In more Bourdieuan terms it is a function of ones position in the field – and therefore the dispositions and capitals that correlate with this position – all of which provides those in such positions with the ability to impose their perspective via encoding it in the evaluative procedures of ‘quality assurance’. A corollary of this is that Goldenberg’s definition also highlights who or what is subordinated to these measures and therefore, the perspectives that are neglected. From a socio-analytic perspective the flexibility and contextual utility suggested by Goldenberg’s definition is not a flaw: it reflects the reality of Quality of Care discourse.

Rather than taking this to undermine Quality of Care and the bureaucratic processes that seek to assure it, we might accept it as an unavoidable consequence of the modern management and governance of health care. Thus rather than question ‘Quality of Care’ *per se* we might offer more specific critiques that focus on its use, and the effects of its use, within particular contexts. Goldenberg’s discussion makes clear that we should not conflate the delivery of care with processes or procedures to ensure its quality and that both can be distinguished from the theorization of care offered by feminist ethics, something that should not be mistaken for a singular or fully unified project [14]. Furthermore, whilst the social organization of health care can, and sometimes does, militate against the caring dispositions of professionals [8], it would be facile to suggest that any and all bureaucratic procedures or managerial processes should be abandoned because of this; a lack of proper managerial oversight will also lead to failures in care. Even when a professionally endorsed procedure becomes the target of criticism, such as in the case of recent controversy about the Liverpool Care Pathway (LPC), there is no argument that a commonality of approach is, in itself, undesirable [15].

What is required is a more sophisticated understanding of the relationship between the front-line practices of care and the way(s) in which they are managed. We must consider the material and social impact of particular rules, guidelines or management structures that act to condition and recondition professional practices. This consideration should go beyond explicitly stated aims and objectives to encompass the broader consequences or practical effects of the policy. Like any form of surveillance the collection of data in the context of ‘New Public Management’ (cf. [16]) is subject to Merton’s ‘law of unintended consequences’ [17]. It is particularly important to consider the collection of ‘Quality of Care’ data and the relationship between this activity and the actual delivery of care. The assumption underpinning such data collection is that it can proceed in such a way as to leave the practice it intends to evaluate untouched. However, consider reported attempts to ‘massage’ or otherwise manipulate records concerning treatment times at NHS Accident and Emergency Departments [18,19]. Recording the time it takes for patients to receive attention does not leave practice untouched when front-line staff have been made aware, that: 1) particular waiting times are acceptable and others are not; and 2) waiting times will be determined in a particular way. From an ‘audit culture’ perspective the collection of data about a particular practice has tangible and immediate effects [20]; it is fallacious and potentially harmful to dichotomize frontline practice and the managerial structures that command, control and facilitate it. Rather, we should reflect critically on the nature of the picture being constructed by such data and, in so doing, attempt to develop an account of the relationship between practice and the management of practice, or between care and Quality of Care, that will facilitate a more fruitful - mutually engaging and enlightening – dialogue, one that engages in the holistic promotion of the caring endeavor.

To conclude this section, whilst the moral or normative sense of care resists easy definition, and this resistance has consequences for our assessment of health care, the need to define ‘Quality of Care’ is unavoidable; it is required if we are to properly govern and organize our health care services. Given this state of affairs we should remain alive to the potential for misalignment and conflict between, on the one hand, professional practices and delivery of care and, on the other, the management of those activities at both the meso and macro levels.

### The unintended consequences of bureaucracy

In his classic article, ‘The Tyranny of Light’, Tsoukas [21] argues that the pursuit of information can undermine, rather than enhance, our knowledge of a particular practice. The supposed objectivity of administrative and bureaucratic records, the instruments of governance, has brought about the dissolution of perspective and allowed an increase in ‘data’ to be mistaken for an increase in insight and understanding. Tsoukas identifies two problematic assumptions: first, that information increases the transparency with which we understand some object; second, that such information can be used to control or (re)engineer that object. Against these assumptions he argues that the logic of governance dictates: “that which is measurable, standardizable, auditable *is* measured…[such measurements come]… to stand for, to represent, the phenomenon at hand” [21]. The implication is that what is not measurable, standardizable, auditable is not measured and so cannot be taken as standing for, as representing, the phenomenon at hand. This is not simply a facet of the ‘objective’ and ‘subjective’ aspects of the target domain; it is certainly possible (although may not necessarily be desirable) to subject the latter to forms of standardized assessment (cf. [22,23]). Rather it is about the nature of bureaucratic representations and the degree to which they can be considered as ‘standing for’ the object, or phenomenon represented. Tsoukas offers a particularly apposite example:

[T]he quality of teaching (an inherently ambiguous notion) tends to be formally ascertained by the quality of the procedures that are thought to lead to good teaching. Procedural ideals of performance represent (and thus reconstruct) our understanding of quality. Notice, however that, as mentioned earlier, ‘quality in teaching’ is nowhere to be seen in the information gathered - it rather needs to be inferred from it [21].

Accounts of ‘Quality of Care’ and the administrative procedures - which is to say bureaucracies - constituted to assess it are similarly and inherently ambiguous. Quality of Care is formally ascertained by the quality of the procedures that are thought to lead to or reflect the delivery of good care as it is inferred from the information gathered by and about these procedures^l^. The information is generated by managerial, administrative and bureaucratic procedures. These procedures shed light on (but do not simply reflect) specific aspects of practice: those that can be codified and therefore recorded. Other aspects are not only left in the shadows but are actively pushed into the shadows as a result of the light being cast^m^. This can be most clearly seen if we consider the reflexive consequences of audit as a mode of management, evaluation and assurance or control [20]. Once something – or *somethings* – become an indicator of performance then those whose performance is being assessed often come to focus on the indicator(s) altering the way in which they accomplish the practice as a whole. For example, it has been suggested that education has become overly focused on ‘teaching to the test’. In health care we have attempts to manipulate, or otherwise circumnavigate, the collection of information used to assess the Quality of Care^n^. More subtly, procedures put in place to support the collection of data relevant to the Quality and Outcomes Framework in general practice, such as structured electronic templates for chronic disease management, have been shown to profoundly influence and shape the conduct and meaning of chronic disease reviews [24]. The fact the practices of governance have reflexive consequences for the practices being governed demonstrates the second of Tsoukas’ points. If social practices are affected by attempts to audit them then the picture generated is not only a poor representation of practice (at the very least the data is often out of date) but the assumed primary virtue of this information, its decontextualized nature, is undermined. If the reflexivity of the social world means that it reacts to our attempts to represent it then our ability to govern on the basis of ‘objective’ information is questionable. Bureaucratic records, the levers of governance, do not produce, or ‘archive’, collections of disinterested facts, or archives of neutral measurements. On the contrary, they are the inscriptions of a studied, and practiced, *disinterest*.

This suggestion opens the door to a number of well-rehearsed critiques of bureaucratic managerialism. In the hands of some this has led to calls to bring the rule of ‘the bureaux’^o^ to an end and predictions of a ‘post-bureaucratic’ era [25]. However, like du Gay [25], we do not consider ‘bureaucracy’ to be a singular phenomena but a “diversely formatted organizational device” (2005:1, 3). It, and paperwork more generally [26], can be considered an ineliminable *technique* in the conduct and organization of contemporary social life, particularly the social life of institutions. It is a technology and one that structures the social world(s) we inhabit. The idea of a bureaucracy cannot be considered good or bad *in itself*, and therefore we should, in each case, consider the inherently normative character of its socially structuring function and the potential moral implications - both intended and unintended - of that structure. As such the idea of a ‘bureaucracy’ cannot be subject to any simply moral evaluation, tempting as it is. Instead we might consider particular instantiations to be morally problematic, particularly those that can be described as overly *bureaucratic*. A *bureaucratic* bureaucracy embodies “an instrumental rationality through which technical questions become split from ethical and aesthetic ones” [27].

As Kafka suggests “[w]e tend to condemn bureaucracy and bureaucrats when better explanations elude us” [26]. At the very least we ought to look for better or, perhaps, fuller explanations before denunciating such an easy target. We should consider the way Quality of Care bureaucracies are constituted and, subsequently, practiced or ‘put into practice’. Which is to say that we should consider the way Quality of Care procedures are *implemented,* how the data produced is *evaluated*, and how these evaluations are subsequently *utilized*. Furthermore, we should be prepared to do so in an ongoing manner. One might start with the idea that health care practices and the *care* they deliver are subject to a set of managerial process that are overly reliant on a form of bureaucratic rationality. Such rationality deploys a set of techniques for the ‘disinterested’ (see below for further discussion of this term) objectification of practice. Insofar as the social organization of health care is a prerequisite for the delivery and promotion of good care and the identification of substandard care such objectification has a good deal to offer. However, bureaucratically rational frameworks can also structure practice in such a way that the provision of excellent care is discouraged. The objectification of practice is reductive as the focus is not on ‘care’ - the intersubjective interplay between those who care and those who are cared for – *per se* but on measure of Care Quality. Certainly the reduction is, to a degree, a necessary and unavoidable consequence of bureaucratic objectification. Nevertheless the objectification of care has, at minimum, the potential to structurally disincentivize the delivery of health care with *care*. Using the Bourdieu’s notion of symbolic violence the next section explores this possibly further.

### Bureaucracy and the potential for symbolic violence

Given it is a socio-historically variable phenomena or, as du Gay has it “a many-sided, evolving, diversified organizational device” [25], no singular conception of bureaucracy can be entirely adequate. If we are to extend our analysis of the disjunction between care and Quality of Care, some greater specification is needed. Furthermore given that this organizational device is variable, ineliminable and cannot be subject to a blanket moral disapproval then we can only seek to subject it to socio-analysis and critique^p^. Using the critical potential offered by Bourdieu’s notion of symbolic violence this section offers a characterization of the particular bureaucracy involved in the organization, management and governance of UK health care [28].

As Clarke [29] points out the political landscape of the UK is such that the associated bureaucracies of its government have been fundamentally (re)configured by the ideology of ‘New Public Management.’ A consequence has been the development of evaluative processes and bodies, including the Care Quality Commission, which attempts to ensure or assure (through a process of evaluation) high quality care. These evaluative institutions are a significant development in modern government’s audit culture and central to the UK’s current ‘arms-length’ approach to the delivery and management of public services [29]. This approach to accountability in the public service sector suggests that bodies - such as the CQC - are something more than *evaluators* of the Quality of Care; they are *regulators*. Given this role we should acknowledge that the process of evaluation - and not simply its results - are effective in shaping practice. As institutions are required to actively produce the data required by regulators, managers and practitioners become aware of the data’s *symbolic* meaning. Furthermore, the CQC is granted the authority to audit and evaluate the delivery of care and, in so doing, to ‘operationalize’ and apply the (politically) working definition of ‘Quality of Care’.

The exercise of authority involves the exercise of what Bourdieu calls symbolic power and, therefore, an inherent asymmetry. However, we should not assume that the existence of such asymmetry is necessarily problematic; the existence of symbolic power and its variable distribution is a fact of social life. Indeed, in this context, it is important to recognize that “the structural and symbolic power wielded by doctors is legitimate, socially conferred and indispensible for help and healing to occur” [30] and we might think similarly for the management and governance of health care. Furthermore, once we have recognized the operations of symbolic power then we might concern ourselves with the way it is being exercised and reflect on whether or not its use can be characterized as involving ‘violence’ or domination. Given its connection to language and knowledge [31] we cannot escape it and we should, pace Foucault’s conception of power more generally, see it as a productive phenomena. Nevertheless, as a form of power, there is the potential for repression as well as production and we should therefore attend to the possibility of what Bourdieu calls ‘symbolic violence’^q^.

The notion of symbolic violence might seem unduly provocative. However we are not suggesting that Quality of Care bureaucracies are, in fact, symbolically violent. Rather we are suggesting that, whether physical or symbolic, the exercise of power always has the *potential* for violence – it is always *possible* for our activities to involve the domination of others. Our view diverges from the Bourdieuan orthodoxy, which would appear to suggest that symbolic violence, and not just symbolic power, can be both legitimate and illegitimate. For example, Bourdieu appears to consider pedagogy as *always* involving symbolic violence [32]. However given that pedagogy is unavoidable, particularly insofar as tacit pedagogy is implicated in human social development (socialization), then it would seem violence is unavoidable. Such a position means that we must discern ‘good’ and ‘bad’ forms of violence. Instead we think that we should distinguish between good and bad forms of symbolic power and consider the latter to be symbolic violence. Thus we prefer to consider education as involving the exercise of symbolic power. As such it is not necessarily violent but may dominate rather than emancipate. Similarly Quality of Care bureaucracies necessarily involve the exercise of symbolic power. The challenge is to ensure we consider the way this power can be legitimately exercised, in the interest of whatever ‘good’ is at hand (in this case the good of care), and to remain alive to the possibility that it might become ‘violent’, illegitimate, and act against the good at hand (which, in this case, would involve the structural domination of health care professionals and the practice of care).

Expanding on Goldenberg’s view of the importance of ‘*relevant stakeholders*’ [11] we can see Quality of Care discourses are not only constructed by distinguishing between who is, and is not, considered a *relevant* stakeholder and, subsequently, by differentiating between those who are *least relevant* and those who are *most relevant*. The consequences of such distinctions will fundamentally alter the Quality of Care discourse and those with the symbolic capital to make, and enforce, such distinctions will dominate the Quality of Care discourse. In this way a particular point of view will be encoded within the symbolic processes and used to evaluate the actual practices of health and social care. Furthermore, in order to enquire into the Quality of Care in specific instances it is inevitable that a bureaucracy procedure will be used and will, therefore, impose a pre-existing and pre-constructed perspective on the practices being evaluated. Through the collection of ‘objective’ (or ‘objectified’) data in accordance with a particular symbolic structure an administrative, or symbolic, representation of the practice will be produced.

Thus, what we know about the Quality of Care, both ‘in theory’ and ‘in practice’ can be seen as part of a struggle over ‘methodology’. A Quality of Care methodology is not simply neutral and objective but *productive* and *world-making*;^r^ it brings something new into existence, namely a declaration regarding the *quality* of the care being delivered. Given that this evaluation (both its results and its implementation) is designed to have a regulatory function then it should be considered a significant locus of symbolic power. Audits are contemporary technologies of evaluation and should be considered part and parcel of the field(s) they render accountable. This is precisely because as forms of bureaucracy, as organizational devices, institutionalized audits act in such as way as to engender ‘audit-ability’. Whether or not it is considered ‘successful’ the attempt to exert control runs counter to the narrative of an audit or evaluation as objective, as leaving that which is audited or evaluated untouched. The institutionalization of an audit culture entails a (re)configuration of the field such that it can be audited and is therefore “easier to regulate in the name of quality” [33,34]. The difficulty posed is similar to that raised by Law in his analysis of social science research methods:

“The argument is no longer that methods *discover* and depict realities. Instead, it is that they participate in the *enactment* of those realities. It is also that method is not just a more or less complicated set of procedures or rules, but rather a bundled hinterland” [35]^s^.

The CQC is part of a ‘bundled hinterland’ and its distance is, in part, created by the methodologies of audit, something that requires what Herzfeld calls ‘bureaucratic indifference’ [34,36] and Bourdieu would call (bureaucratic) *disinterest* [37]^t^. However, in creating a space within which symbolic power can be exercised bureaucracy becomes *distanced* from practice. This creates the potential for bureaucracies to not only “slip from the model of reality to the reality of model” [38] but for structurally embedded procedural imperatives to become privileged over the ends of practice [39]. It is at this point that the potential for symbolic power to become problematic emerges. The distance that the practice of disinterest creates generates the conditions within which bureaucratic exercises can become symbolically violent and therefore come to place structural constraints on the caring practices they are supposed to promote.

This view accords with Strathern’s suggestion that “current practices of audit and surveillance are far from ‘indifferent’; on the contrary they present the face of obsessive concern (care/interference)” [34]. The terminology of ‘obsessive concern’ and ‘interference’ is suggestive of intrusion, violence and domination and, furthermore, the ability of audit to exert its influence is predicated on its methodology, its ‘objectivity’, something that is not produced through indifference *simpliciter* but through a *studied* and *practiced* indifference, or Bourdieu’s notion of *disinterest*. The posture of disinterest expresses a particular kind of interest, one that values *objectivity*, which is to say a form of *symbolic neutrality* and is, therefore, a function of an underlying symbolic capital and power. It is through the adoption of a disinterested stance that *audits* (auditors and those who promote the audit as a style of evaluation, management and *quality* control/ assurance) purport to care. This is assumed to be necessary because such disinterest produces an ‘objective’ or, more accurately, ‘objectified’ picture of health care. This is held to be the first step in a process of ‘continuous improvement’ or ‘total quality management’. Bodies like the CQC are invested with the political authority to name, categorize, pronounce judgment and determine social reality - the ‘facts’ of the matter - precisely because they appear to express a disinterested form of interest, they appear to be objective [32]^u^. However, symbolic domination is not merely a function of these pronouncements but of the way those judged anticipate and respond to these pronouncements and the way in which they are produced. Bourdieu and Boltanski suggest that:

“Symbolic domination really begins when the misrecognition (*meconnaissance*) implied by recognition (*reconnaissance*) leads those who are dominated to apply the dominant criteria of evaluation to their own practices”.

([40], cited in [41] authors translation).

In the case of Quality of Care audits or evaluations one should not simply blame those who are dominated for their own domination. However, they are ‘complicit’ in the way that the supposed *measures* of Quality have become reiterated – recognized and misrecognized - as *targets*^v^. This slippage is, in part, a function of the law of unintended consequences, but one that has come to be embedded in the logic of New Public Management; what was once a systemic problem, and an organization vice, has been turned into a managerial strategy, and an organizational virtue^w^. Quality of Care evaluations have come to have priority in the actual delivery of care and therefore methodological indicators - measures – of care quality come to be seen as targets – substantive facts about care quality. The actual practice(s) of health care have become subordinated to ‘Quality of Care’ and, rather than being responsive to patients, professionals are increasingly required to respond to the imperatives of the evaluative bureaucracy invested with the symbolic power to pass judgment. The violent exercise of symbolic power is essential to the development and maintenance of this dynamic; it is a consequence of allowing a form of bureaucracy to become a management style, the function of which is predicated on having the symbolic capital required to monopolize, which is to say *dominate*, the process. This all but guarantees that the exercise of symbolic power will entail symbolic violence and that frontline practices are subject to the symbolic domination by the organizational mode meant to facilitate it.

## Summary: caring about quality of care

At the outset of this paper we distinguished between the moral concept ‘care’ and the professional practice of health care. We suggested that the latter can be done with more or less of the former and that excellent health care is associated with more, and not less, moral care. Concern for the Quality of Care is concern that health care is done with care; it is a moral concern. However, any attempt to evaluate the Quality of Care in particular cases or institutions can only gesture towards an evaluation of ‘care’ as a moral ideal. The practicalities of bureaucracy, audit, evaluation and ‘quality assurance’ methodology mean that whilst we can construct symbolic representations of the Quality of Care predicated on the practical delivery of health care, care itself remains a frontline task that can only be guaranteed by those who actually deliver it, their ethics and professionalism. The Quality of Care discourse finds its main usefulness in the management and organization of health and social care. As such it can contribute towards the provision of care but cannot guarantee care as a moral phenomenon. Furthermore, the law of unintended consequences means that institutionalized auditing processes of such bureaucracies may actively militate against care as a moral practice. Paradoxically the institutionalized goals or targets created by bureaucratic processes of ‘assurance’ have a not insignificant potential to displace the goals they are seeking to assure. As a symbolic practice Quality of Care evaluations have the potential either to dominate or emancipate the *actual* practice of care. Arguably Quality of Care evaluations appear to be subjecting contemporary health care practices and practitioners to a greater level of domination than emancipation. This is, at least in part, a function of measures becoming targets, something that has unintended consequences, not least of which is the production of perverse incentives. The symbolic and structural domination of health care professionals is such that they may become overly responsive to concerns and targets rooted in Quality of Care discourses and frameworks at the expense of being responsive to the needs of patients for care.

If this is the case then it is imperative that we collectively consider how ‘our’ political expectations promote symbolically violent forms of bureaucracy and audit. Furthermore we should engage with the unintended consequences of bureaucracy and the potential for symbolic violence. Whilst a comprehensive response is beyond the scope of this essay we can offer some suggestions predicated on the notion of care as a moral, or *ethico-political*, concept. In the first instance we suggest that it is necessary to rethink our understanding of Quality of Care as an auditable phenomena. If we regard care as involving emotional investment, or an activity that involves the repair of our ‘world’ such that we can live well [3] then it is not something that can be subject to a comprehensive audit. It cannot be considered *fully accountable* to any organizational device or bureaucracy, and any attempt to render it fully accountable will founder. If we want care of a certain quality there is a point at which we have to trust the professionals who would care for us. This does not mean simply letting professionals (or professions) ‘off the hook’ but explicitly revisiting the notion of the profession as vocation, with which comes a set of rights, privileges, duties and obligations. This might point to a need for bureaucratic reorganization and reorientation. If we were to reimagine Quality of Care evaluations then we might think if them as caring *for* rather than simply caring *about* the delivery of good care, and this would necessarily include a need to care for the professionals charged with delivering care. Such a re-orientation would bring the actions, judgments, orientations and dispositions of practitioners in particular caring contexts to the foreground. This would mean abandoning the objectivity of ‘studied disinterest’, something that allows the CQC to care about practice from a distance. It would mean recognizing the complexity, ambivalence and shifting tensions [10] of social reality and the inadequacies, or limitations, of any methodological evaluation, particularly prescriptive methodological evaluation of the sort required for the *enactment* of a reality that allows measurements to be transmuted into targets. We need to ‘unbundle’ the hinterland of the CQC and open it up to the kind of persistent tinkering that, for Mol and her co-authors [10], signifies the specific modality of care, and the *care-ful* pursuit of the good. Such tinkering presents a challenge to the procedural objectification of practice inherent in audit and bureaucratic rationality, demanding that they exhibit an ‘attuned attentiveness’ and adopt an ‘adaptive’ posture [10]. Such a stance would not only militate against mistaking Quality of Care models for the reality of care itself, but act against the potential for procedural imperatives to become privileged over the ends of health care. Rather than ‘adapting’ practice to bureaucratic structures, as in the case of patients waiting in ambulances outside of casualty, we should become attuned to the limitations of procedurally generated data and attend to the way in which this data is used. In this way the assurance of care quality becomes a form of ongoing experimentation and, ideally, one that encompasses the ability to reflexively respond to changes in practice, some of which will be produced through Quality of Care activities themselves.

The idea of ‘persistent tinkering’ connects with Tronto’s suggestion for ‘creating caring institutions’ [42]. It also furthers her perspective that the moral concept of ‘care’ presents a challenge to the traditional boundary between morality and politics [3]. It suggests that the CQC should itself become a caring institution, one that cares for Quality of Care and those who deliver it. As such it should not simply implement political defined programs of audit but address the implications and consequences of such evaluations. One way of doing this would be to more actively involve health care professionals in their own evaluations and in the constitution of the evaluative process as a whole. It might also suggest that we abandon, or at least relax, the methodological rigidity of Quality of Care frameworks, the basis of impartial audits. Whilst this rigidity facilitates institutional comparisons, something that is not necessarily objectionable, it does so to such a degree that we have become encouraged to produce league tables. This approach does little to *care* for our system of health care either as a whole or in respect of its particular institutions. Loosening this rigidity would facilitate the kind of ‘persistent tinkering’ that, “in a world full of complex ambivalence and shifting tensions” [10], Mol, Moser and Pols consider to be indicative of *good care*. Whilst this suggests we should abandon an inflexible approach to Quality of Care audits we are not suggesting that we abandon a methodological approach to such evaluations or that we engage in a form of methodological anarchy or, at least, not outright. We should, however, open our evaluative methodologies to a form of ‘persistent tinkering’ consistent with Bourdieu’s conception of reflexivity [43]. Given that what is under discussion is not social scientific research but, broadly speaking, the use of social science methods in the process of social life then we might consider the institutionalization of ‘reflexivity’ as a part of this process rather than, merely, a function of the researcher or, in this case, the auditor.

## Endnotes

^a^In this paper we capitalize the term ‘Quality of Care’ to indicate that we are talking about the discourse and specific sub-disocurses as well as the formal evaluation of care to which such discourses have given rise.

^b^As will become clear in our discussion of Quality of Care and the Care Quality Commission (CQC) the division between the latter two levels – managerial (meso) and political (macro) - is increasingly blurred. This is because ‘arms-length’ bodies are designed to inoculate the political sphere from its responsibilities and its accountability, and so the supposedly autonomous managerial domain becomes politicized by the intrusion of these same bodies [29]. This fact is borne out by the ‘political’ nature of Quality of Care discourses (see below discussion below).

^c^See Mol [1] on the notion of ‘cure’ as ‘care.’

^d^The metaphor of accounting, and the way it shapes of ‘accountability’ [33] is a vital part of our contemporary audit culture and the rhetoric that maintains it [34]. Indeed Strathern sees it as fundamental, suggesting that “accountancy [has] become linked to a more general idea of accountability, and with it an expansion of the domain of auditing” [44]. Furthermore the preference for accountability over responsibility was, Kafka [26] suggests, central to the creation of ‘paperwork’ and bureaucracy as *the* organizational form of the modern nation state.

^e^There are, of course, plenty of other definitions and discussions of care to be found in the literature on feminist ethics. Held, for example, considers it to be “both a practice, or cluster of practices, and a value, or cluster of values” and distinguishes this from the characteristics (dispositions) of caring persons [45]. Furthermore, such conceptual diversity should be seen as a strength of feminist ethics, a mode of thought that perceives the value in being a collective and dialectical endeavor [46]. This whilst this essay adopts Tronto’s perspective on ‘care’ we do not seek to be dogmatic in our attachment to it. We thank Professor Johnathan Herring for his comments regarding alternative models of care.

^f^Of course, in practice, the existence of such motivation will vary and may be more or less inchoate. It might also be subject to erosion.

^g^Much of Menzies Lyth’s other work is also highly relevant. In particular see Volume 1 of her Selected Essays, ‘Containing Anxiety in Institutions’ [47]. Also see Jocelyn Cornwall’s conference presentation ‘Words, deeds and structures: patients’ experience and human factors in health care delivery and policy’ (http://www.cumberlandlodge.ac.uk/Programme/Recent%20events/Quality%20in%20Healthcare). The paper was delivered to the 2013 Cumberland Lodge Colloquium ‘The Many Meanings of Care’ co-organized by the authors of this paper, further information and resources are available from the legacy site: http://www.cumberlandlodge.ac.uk/Resources/CumberlandLodge2011/Documents/Programme/Reports/Quality%20in%20Healthcare%20report.pdf.

^h^In her discussion of what she means by ‘logic’ Mol draws on the notion of a discourse within which “words, materialities and practices hang together in a specific, historically and culturally situated way” [1]. Her analytic use of the term is an attempt to get at the “rationality, or rather the rationale, of the practices” [1] at hand, namely health care. Below we turn this idea - of the embedded logic of a practice - on the practices bureaucracy, management and *governance*.

^i^To say that error and even neglect are an ineliminable part of everyday life and, therefore, of health care does not to invite us to adopt a fatalist approach to their existence or, worse, particular instances of their existence. Rather it is to acknowledge the ideal notion of care operative in this context and the problem with thinking it is always and everywhere achievable. Such a view would not only be an act of individual and collective hubris but to misunderstand the nature of the concept being put forward and the complexity of (human) being. Thinking that neglect and error are fully eliminable is akin to thinking that no hospital can (or should) perform at a ‘below average’ level.

^j^As discussed further below the particular formation is that of ‘New Public Management’ [16].

^k^Our use of the term appeals to its original sense, as something intimately associated with the administration of government and what, in the UK, is called the Civil Service, see du Gay [48] and Kafka [26].

^l^As a reviewer of this article points out it is interesting that the CQC is focused on procedures rather than outcomes. In part this is because healthcare has managed to resist the imposition of ‘league-tables’, something that can be contrasts with similar quality assurance projects in other domains such as education. This is likely due to the greater institutional and political power of health care and, specifically, the medical professional to resist the external imposition of evaluative assessments as compared to that found within education and wielded by teachers and academics. Thus, a focus on outcomes cannot be positioned as an alternative mode of measurement or evaluation as they remain determined by bureaucratic and procedural means. Furthermore, it is unclear if, or for how long, this state of affairs will continue. Given current developments it is likely that, in one shape or another, health care will be subject to outcome evaluations. We thank Professor Søren Holm for pushing us to make this point clear.

^m^It is worth drawing the readers’ attention to Strathern’s essay ‘The Tyranny of Transparency’ [49]. Here she builds on Tsoukas and considers audit as a process of ‘making thing visible’ and what such visibility might conceal.

^n^Consider the 2008 report on the fact that some patients were being kept in ambulances as Accident and Emergency waiting times, something subject to evaluation, only began once patients exited ambulances and entered hospitals [18,19].

^o^As Kafka points out that the creation of Bureaucracy as a form of government has its roots in the French Revolution and was, literally, the invention of a new form of rule not “by the many [democracy], the few [aristocracy], and the one [monarchy] but … by a piece of office furniture” [26].

^p^The term socio-analysis can be considered a “collective counterpart to psycho-analysis … [that] can help us unearth the *social* unconscious embedded into institutions”, [50] cultures and societies. It is often used by Bourdieu and indicates the potential his conception of sociologically reflexive critique has for resistance and emancipation. Socio-analysis introduces an ethico-political dimension to the social sciences, to sociology and to sociological critique [51].

^q^In what follows we diverge from the Bourdieuan orthodoxy, which suggests symbolic violence, and not just symbolic power, can be both legitimate and illegitimate. Bourdieu develops his notion of symbolic power through an analogy with physical violence [50]. In particular he focuses on the way the state seeks to monopolize the exercise of physical violence. We demur from his analysis and suggest that the state seeks to monopolize the use of *legitimate* physical force. However, at least in part, it does so through legally, which is to say symbolically, defining what is and is not legitimate physical force. Thus the point at which *legitimate* force becomes *illegitimate* violence is determined by the symbolic power invested in the state. What counts as legitimate and illegitimate is determined by reference to the symbolic structures of society and, therefore, through the exercise of symbolic power and, implicitly or potentially, violence. Thus, as Bourdieu suggests, the problem of symbolic violence is at its most insidious when it is misrecognized as legitimate.

^r^The ‘world-making’ properties of symbolic capital and power is an essential facet of its potential for both violence and emancipation [52,53].

^s^Bourdieu also connect the problems of bureaucracy to those of the social science suggesting that “All the issues raised about bureaucracy, such as those of neutrality and disinterestedness, are posed also about sociology itself – only at a higher degree of difficulty” [37].

^t^Bourdieu contrasts the terms indifference and disinterest. The latter is actually a particular kind of interest. As such it can be part of what Bourdieu calls a field’s *illusio* and, therefore, a strategy for success. Consider, for example the disinterest of the scientist and the way this is a form of interest rather than indifference. In what follows we refer to disinterest as a kind of ‘studied and practiced indifference.’ However this contrasts with true indifference which is to be understood as a lack of dis/interest. Thus when Strathern, Herzfeld and Shore (see below) use the term *indifference* they do so in accordance with Bourdieu’s *disinterest*. This point resolves the problem being raised, as Bourdieu’s conception of disinterest is a form, mode or style of interest invested with a particular sort of symbolic capital. Such capital is central to the social and symbolic power of bureaucracies, audits and, for that matter science, including social science ([37], cf. [54]).

^u^The fact that the CQC had many of its pronouncements undermined in late 2009 when they were seen to conflict with The Good Hospital Guide, a publication produced by academics working on behalf of a private Company (Dr Foster Intelligence), can be seen as confirmation of this point: social reality is not simply a matter of fact but of power and having the symbolic capital to prevail. As Bourdieu says, “[s]ymbolic power is the power of creating things with words” [53]. In this case the word of ‘Dr Foster’ and the Good Hospital Guide outweighed those of the CQC. See Taylor [55] for an account of this conflict. We would also note that the fact this controversy resulted in a conflict between two different auditors acts to insulate the political sphere from its responsibilities.

^v^Significantly, the point originates in an analysis of monetary policy where it is know as Goodhart’s Law [56].

^w^Of course, whilst little reminder is needed, it is clear that this development has not been entirely successful.
